# Dentinal Structure, as Related to the Theory of Heitzmann

**Published:** 1883-05

**Authors:** Wm. G. Brittan


### Dentinal Structure as Related to the "Theory of Heitzmann."

Wm. G. Brittan.

A tooth, as a whole, is known to every one. But in its formation a great variety of peculiar properties, physical and chemical agencies, all so combine as to render the study of its minute structure quite complex. So much so that probably no tissue of the human body presents more or greater difficulties to the histologist than that of the dental organs.

Instrumental investigation, when conducted with high powers, is somewhat unreliable and attended with great difficulties, forcing a reliance upon appearances which often lead to erroneous conclusions.

In our everyday experiences when brought in contact with objects—with which we are unfamiliar—we are always careful how we pronounce their identity upon the evidence of sight alone, but require the evidence of other senses before we are positive. Herein lies the one great difficulty in the pursuit of these investigations. We are too much dependent upon the sense of sight, which is of all the senses the one the most liable to be deceived. As a consequence histologists disagree and theories conflict.

Dental histology is a science of comparatively recent growth, and, although we are to-day somewhat in advance of the earlier writers, there are yet many points of vital interest in the dark, as also some recent theories that would seem to require further elucidation before they should claim our acceptance.

One to which it is more particularly the purpose to call attention is what is known as the "Bioplasson," or the reticulated structure of living matter as distributed in the teeth.

This theory may be regarded as the direct outcome of a criticism directed by the late Max. Schultze against the cell theory of Schwann. This criticism was, I think, published in 1861, in which we are told there does not exist a tissue in the human body built up of cells as taught by Schwann, but what formerly was thought to be a cell is now recognized as a jelly-like mass with a reticulated structure. The nucleolus being connected with the walls of the nucleus, and this again with granules of the protoplasm by fine threads of living matter, forming a net-like structure—this structure being continuous throughout the whole body.

In 1873 we learn that Dr. Heitzmann extended this discovery into bone tissue and the dentine. The lacunæ of bone tissue being the spaces in the glue-giving basis substance. Each of these spaces being occupied by a protoplasmic body with a distinctly visible reticulated structure—which we are told must be regarded as the living matter of the protoplasm—while the fluid contained in the meshes of living matter does not possess the properties of life.

As you are all familiar with this theory as it regards the dentine, a brief summary will only be required in this structure. In the dentine he found that fibers of living matter from the "odontoblasts" of the pulp not only enter the dentinal canaliculi but also extend beyond the boundary of the dentine into the cement substance of the enamel.

Also, that the basis substance of the dentine is in all directions pierced by these fibers of living matter forming a net-like structure.

Many writers, and probably observers, have, from time to time, corroborated this theory, and from the present tone of our literature, I think the statement is justified that it has already become an established doctrine in dental morphology. Whether it can reasonably maintain that ground is to me a matter of great doubt. I do not say that these structures do not exist, but I say the *truth* of the theory has never been demonstrated, neither can it be, notwithstanding all the present improved facilities for investigation.

There seems to be some confusion in terminology among writers upon this subject. The term *protoplasm* is now so often used that it is time it should be restricted to some definite idea.

*Bioplasm* is a term well applied to express matter in its living state, while *protoplasm* should be restricted to the material itself.

Living matter or bioplasm, according to Mr. Huxley and others, is a "semi-fluid substance, being a compound of the chemical elements," and called by him and others, the "physical basis of life." It does not freeze at 32° F. It is in a constant state of molecular change, since it is constantly receiving pabulum and transforming itself into formed material. When change takes place from bioplasm into formed material combination occurs, but the formed material is not living matter or bioplasm, although it may have acquired special properties as the elasticity of muscle or the conducting power of nerve tissue.

The life is gone. It is now only a medium through which the phenomena of life is expressed.

As Mr. Huxley expresses it the "peculiar properties of living matter distinguish it absolutely from all other kind of things."

Now, these solid fibres, as they are described to us, have all the characteristics of formed material, having definite form, shape and size, the fibres in one tubuli corresponding exactly with those in all the others. They also stand any amount of rough usage, such as treatment with acids and alkalies. They may be boiled or frozen and can be stained with various mediums, the same as other tissues.

Therefore, if they exist at all, they exist as formed material, created for special purposes and endowed with special properties. They are a product, and are no more the living matter of the protoplasm than any other tissue of the body. It has been quite recently claimed to have been discovered that this reticulated structure exists in the blood corpuscle. Why do not these gentlemen, who are so expert with their microscopes, settle the question as to whether the blood corpuscle is, or is not, a nucleated body, or if it be possessed of a membranous covering? These would seem to be the two things most easily decided. Let us examine some of the methods by which these wonderful structures, of living matter are revealed. We all know that in the examination of tissues by the microscope that only those parts are to be distinguished which refract light differently from the surrounding parts.

Thus it happens that fibres and cells, which are embedded in tissues or fluids, the refractive power of which is the same as their own, cannot be perceived even with the best glasses, and artificial means must be employed to render them visible.

These may be mechanical, effecting separation or isolation, or chemical, which dissolve the connecting material or act differently upon it than upon the morphological elements.

The best artificially prepared specimens cannot, however, supply the advantages of examinations made upon fresh specimens. It is remarkable how these tissues of the teeth may be decalcified, stained, doctored and tortured until the Infinite Source of their conception would fail to recognize them, and still show these infinitesimal fibres of living matter. The transplantationists should draw a great deal of comfort from this theory. Almost everyone who uses the microscope is acquainted with that test object called the *amphiplure pelucida.* The striæ upon this diatom is estimated by Carpenter to be 80,000 to the inch. The resolution of these striæ is regarded as expert work, requiring perfect glasses with complete isolation and perfect illumination.

Now, the supposed dentinal fibres of the tubuli at their base near the pulp have an average diameter of 1-40,000 of an inch; near the periphery of the dentine they would be at least one-half that or 1–80,000 of an inch, these in the basis substance of the dentine and in the cement substance of the enamel are very minute, probably something less than 1–200,000 of an inch. And all these things are seen in a decalcified semi-transparent tissue—in fact, they can be seen in no other way, as isolation of these fibres is impossible. The tubuli may be isolated, but the fibres never—all testimony to the contrary notwithstanding. Because if they exist at all, any known methods, which could be employed for that purpose, would, from the nature of their composition, destroy them as I shall endeavor to show.

As to the real significance of the dentinal tubuli very little is as yet, I think, understood.

Stricker describes three distinct structures—the dentinal canaliculi, the dentinal sheaths, or tubuli, and the dentinal fibres.

The tubuli or sheaths he says, "it is highly probable belong to the category of elastic limiting layers." Neuman considers them to be calcified. Again, according to Stricker, they are a peculiar dense and resistant layer of the matrix, which cannot be destroyed by boiling in strong muriatic acid—constituting the only indestructible residue of the tooth." Of the fibres, he says, "they are really solid and homogeneous." That they easily stain with carmine and possess a remarkable degree of extensibility —so that in young teeth the dentinal cells may be separated a considerable distance from the dentine without rupture. They then appear like "harp-strings" stretched across the interval.

These things, he says, may be seen with a power of 500 linear. Therefore, it occurs to me, that possibly Stricker may have mistaken the tubuli for the fibres, as they can be withdrawn as he describes in young teeth, and as the fibres in this locality are only about 1–40,000 of an inch in diameter, the power used would hardly be requisite to show them.

The statement in regard to the extreme denseness and indestructibility of the matrix substance upon the walls of the tubuli must be incorrect, because no part of the matrix enters into the composition of these structures, and if calcification ever occurs in their walls it takes place long after the solidification of the matrix, an entire differentiation of structure being the result.

It is a well known fact that in almost all examinations of these tissues undeveloped and young teeth have been used. In fact, we are told we should employ no other. As a consequence, therefore, certain conditions may have escaped notice which would have been observed had there been more diversity in the specimens used.

In the teeth of the aged, and in certain pathological conditions, there are indications that the tubular walls become entirely calcified, also, that the inter-tubular canals become filled with calcific matter, in which condition, I conjecture, that in structure they may be somewhat analagous to calcified hyaline cartilage.

This feature of calcification of the tubuli I have also noticed in some teeth, which I have examined, which were lost from absorption of their investments.

From these observations I should infer that where entire calcification of the tubuli occur it should be regarded as pathological.

A more explicit differentiation in the description of these structures would remove much obscurity arising from the misapplication of terms among writers.

As science will not be likely to be thrown off its equilibrium by my interpretations, I may venture to give a view of them briefly.

If I were asked to give an histological description of the dentine I should say it is a *distinctly lamellated* calcareous stroma, very analagous to osseous tissue—in which the calcified expression of the cellular structure of the papillæ is preserved, interspersed with radiating canal-like systems, having no preceptible special parietes, through which run the dentinal tubuli, which are *essentially tubuli* and no other thing—their tubular walls occupying two-thirds of their diameter.

These tubuli are never found calcified, except in pathological conditions. I judge this because in all undecalcified normal specimens the walls of these structures may be saturated throughout their entire length with carmine while the basis substance remains unstained. This characteristic is very marked both in fresh and dry specimens and is a circumstance that most certainly does not suggest the extreme dense structure that is described by most writers, their indestructibility and insensibility to acids being the result of the presence of oleaginous matter, which may be demonstrated by treatment with potash. Through these tubuli run the supposed "fibres of living matter" of which nothing is known except theoretically. The lamellated structure of the dentine is very beautifully illustrated in teeth treated with potash. This characteristic I have never seen described by any of the authors upon this subject, the most of them, I believe, teach that such a condition of structure does not exist in the teeth. It is, however, capable of demonstration beyond the probability of a doubt.

This condition of structure was first noticed by me about three years ago, and as it occurred quite accidentally, was something of a surprise at the time as I had never before had any intimation of its existence. In preparing a tooth for the purpose of showing this structure, it should be placed in a hot solution of caustic potash of a strength of at least 50 per cent, in about fifteen or twenty minutes the cementum may be readily removed, often slipping from the apex of the root in the form of a cap, and by repeated maceration and scaling the lamellæ of the dentine may be removed down to the pulp cavity. I have often removed these lamellæ in sections embracing half the circumference of the root. It has been suggested to me that this condition was pathological, and was the result of interrupted nutrition. This, however, is not so because it occurs in all normal young or adult teeth, besides the lamellæ are of a uniform thickness, so far as I can judge, throughout the tooth. I have never measured them, but from comparative measurements, I judge them to be about 1-12,000 of an inch in thickness. In very old teeth I find their separation more difficult, but it can be accomplished even in such teeth to a limited extent. This structure cannot be seen in cross-sections of the dentine in its unprepared condition, for the reason that the organic and inorganic parts are so intimately blended that the refractive power of the mass is equal in all its parts, but by removing the gelatinous portion, the fact becomes so evident that one trial of the experiment will convince the most skeptical.

I have no reputation as a histologist to lose, but I maintain that a lamellated structure can be proven to exist in the dentine, as a normal feature, beyond the *possibility* of a doubt.

